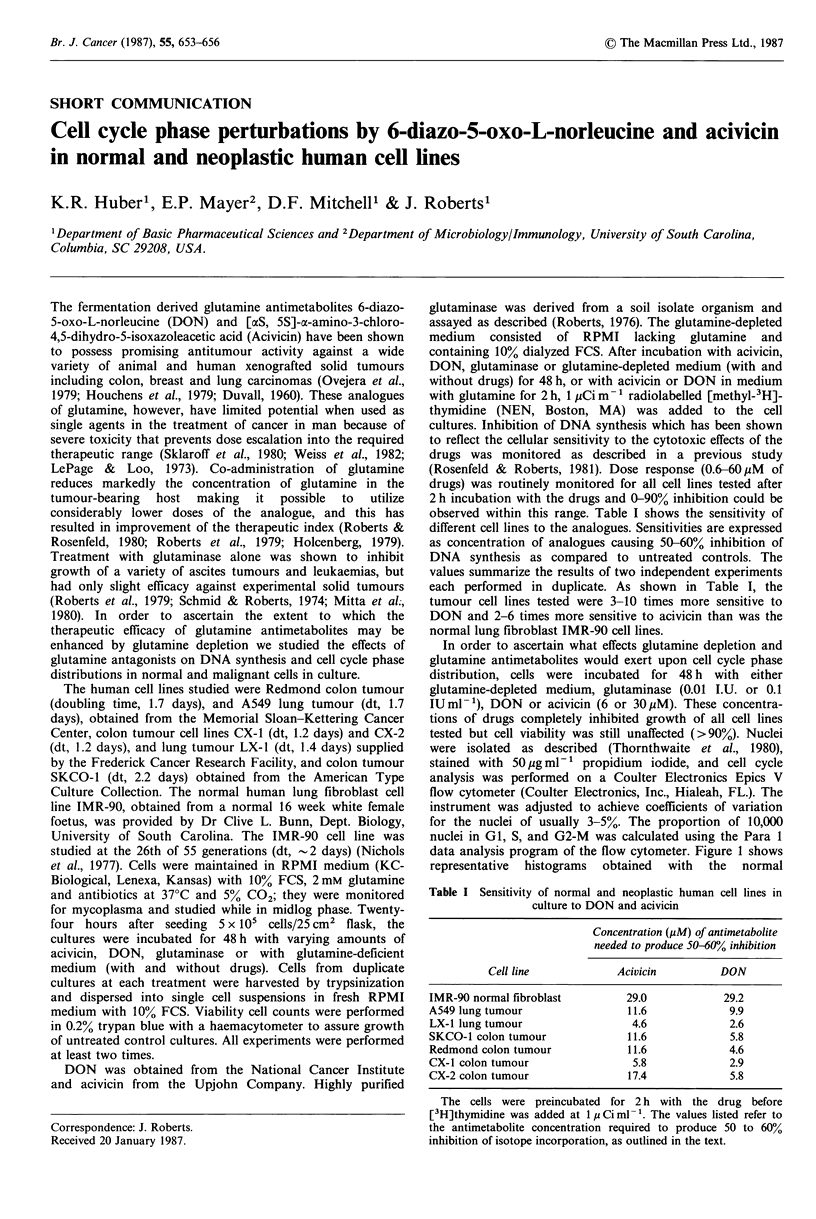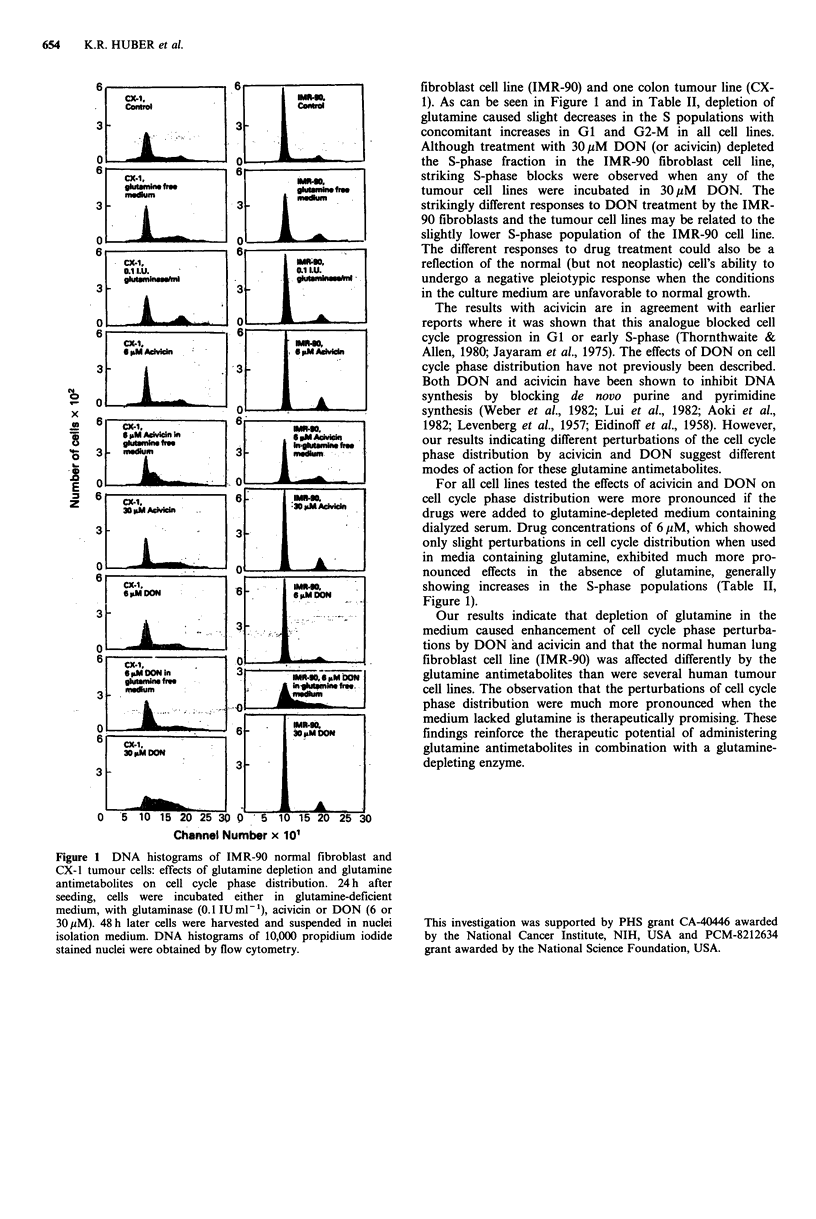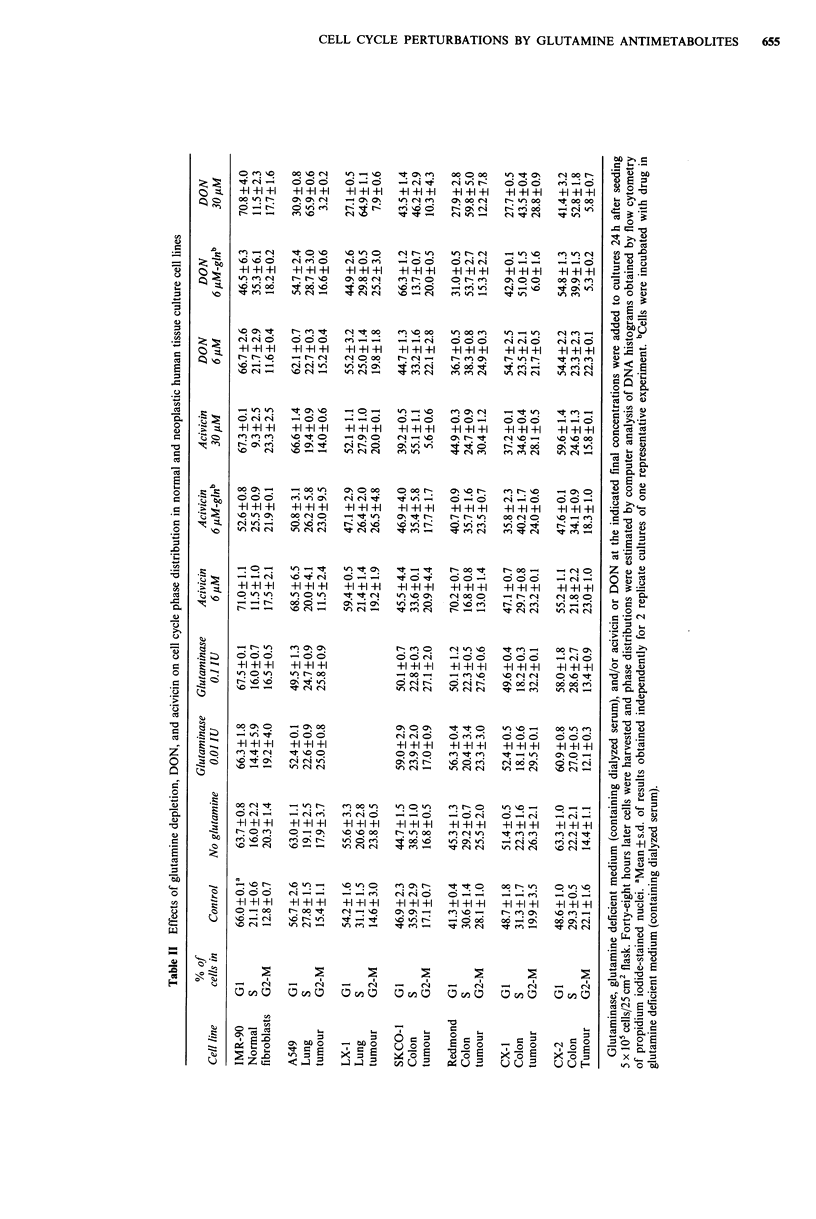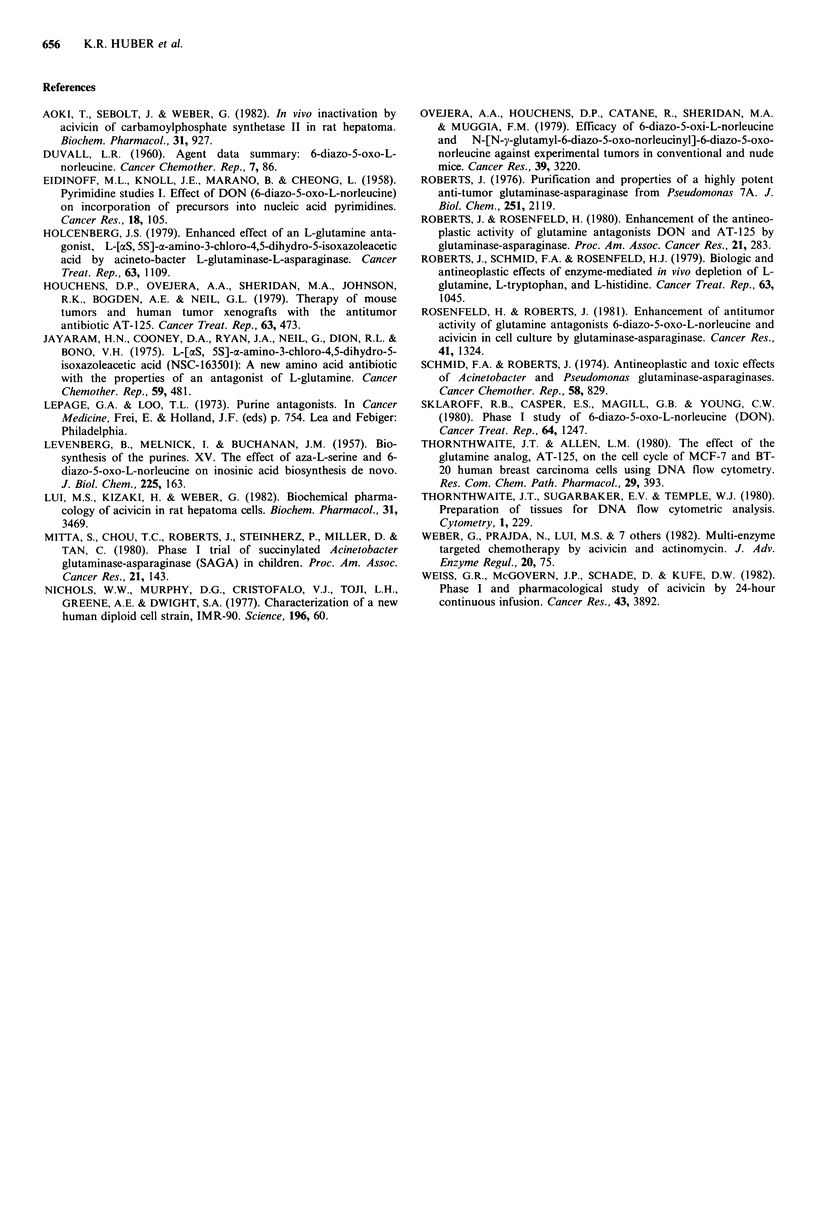# Cell cycle phase perturbations by 6-diazo-5-oxo-L-norleucine and acivicin in normal and neoplastic human cell lines.

**DOI:** 10.1038/bjc.1987.133

**Published:** 1987-06

**Authors:** K. R. Huber, E. P. Mayer, D. F. Mitchell, J. Roberts


Br. J. Cancer (1987), 55, 653–656 © The Macmillan Press Ltd., 1987

SHORT COMMUNICATION

### Cell cycle phase perturbations by 6-diazo-5-oxo-L-norleucine and acivicin in normal and neoplastic human cell lines

K.R. Huber[1], E.P. Mayer[2], D.F. Mitchell[1] & J. Roberts[1]

[1]*Department of Basic Pharmaceutical Sciences and* [2]*Department of Microbiology/Immunology, University of South Carolina, Columbia, SC 29208, USA.*

The fermentation derived glutamine antimetabolites 6-diazo-5-oxo-L-norleucine (DON) and [αS, 5S]-α-amino-3-chloro-4,5-dihydro-5-isoxazoleacetic acid (Acivicin) have been shown to possess promising antitumour activity against a wide variety of animal and human xenografted solid tumours including colon, breast and lung carcinomas (Ovejera *et al.*, 1979; Houchens *et al.*, 1979; Duvall, 1960). These analogues of glutamine, however, have limited potential when used as single agents in the treatment of cancer in man because of severe toxicity that prevents dose escalation into the required therapeutic range (Sklaroff *et al.*, 1980; Weiss *et al.*, 1982; LePage & Loo, 1973). Co-administration of glutamine reduces markedly the concentration of glutamine in the tumour-bearing host making it possible to utilize considerably lower doses of the analogue, and this has resulted in improvement of the therapeutic index (Roberts & Rosenfeld, 1980; Roberts *et al.*, 1979; Holcenberg, 1979). Treatment with glutaminase alone was shown to inhibit growth of a variety of ascites tumours and leukaemias, but had only slight efficacy against experimental solid tumours (Roberts *et al.*, 1979; Schmid & Roberts, 1974; Mitta *et al.*, 1980). In order to ascertain the extent to which the therapeutic efficacy of glutamine antimetabolites may be enhanced by glutamine depletion we studied the effects of glutamine antagonists on DNA synthesis and cell cycle phase distributions in normal and malignant cells in culture.

The human cell lines studied were Redmond colon tumour (doubling time, 1.7 days), and A549 lung tumour (dt, 1.7 days), obtained from the Memorial Sloan–Kettering Cancer Center, colon tumour cell lines CX-1 (dt, 1.2 days) and CX-2 (dt, 1.2 days), and lung tumour LX-1 (dt, 1.4 days) supplied by the Frederick Cancer Research Facility, and colon tumour SKCO-1 (dt, 2.2 days) obtained from the American Type Culture Collection. The normal human lung fibroblast cell line IMR-90, obtained from a normal 16 week white female foetus, was provided by Dr Clive L. Bunn, Dept. Biology, University of South Carolina. The IMR-90 cell line was studied at the 26th of 55 generations (dt, ~2 days) (Nichols *et al.*, 1977). Cells were maintained in RPMI medium (KC-Biological, Lenexa, Kansas) with 10% FCS, 2 mM glutamine and antibiotics at 37°C and 5% $CO_2$; they were monitored for mycoplasma and studied while in midlog phase. Twenty-four hours after seeding $5 \times 10^5$ cells/25 $cm^2$ flask, the cultures were incubated for 48 h with varying amounts of acivicin, DON, glutaminase or with glutamine-deficient medium (with and without drugs). Cells from duplicate cultures at each treatment were harvested by trypsinization and dispersed into single cell suspensions in fresh RPMI medium with 10% FCS. Viability cell counts were performed in 0.2% trypan blue with a haemacytometer to assure growth of untreated control cultures. All experiments were performed at least two times.

DON was obtained from the National Cancer Institute and acivicin from the Upjohn Company. Highly purified glutaminase was derived from a soil isolate organism and assayed as described (Roberts, 1976). The glutamine-depleted medium consisted of RPMI lacking glutamine and containing 10% dialyzed FCS. After incubation with acivicin, DON, glutaminase or glutamine-depleted medium (with and without drugs) for 48 h, or with acivicin or DON in medium with glutamine for 2 h, 1 μCi $m^{-1}$ radiolabelled [methyl-$^3$H]-thymidine (NEN, Boston, MA) was added to the cell cultures. Inhibition of DNA synthesis which has been shown to reflect the cellular sensitivity to the cytotoxic effects of the drugs was monitored as described in a previous study (Rosenfeld & Roberts, 1981). Dose response (0.6–60 μM of drugs) was routinely monitored for all cell lines tested after 2 h incubation with the drugs and 0–90% inhibition could be observed within this range. Table I shows the sensitivity of different cell lines to the analogues. Sensitivities are expressed as concentration of analogues causing 50–60% inhibition of DNA synthesis as compared to untreated controls. The values summarize the results of two independent experiments each performed in duplicate. As shown in Table I, the tumour cell lines tested were 3–10 times more sensitive to DON and 2–6 times more sensitive to acivicin than was the normal lung fibroblast IMR-90 cell lines.

In order to ascertain what effects glutamine depletion and glutamine antimetabolites would exert upon cell cycle phase distribution, cells were incubated for 48 h with either glutamine-depleted medium, glutaminase (0.01 I.U. or 0.1 IU $ml^{-1}$), DON or acivicin (6 or 30 μM). These concentrations of drugs completely inhibited growth of all cell lines tested but cell viability was still unaffected (>90%). Nuclei were isolated as described (Thornthwaite *et al.*, 1980), stained with 50 μg $ml^{-1}$ propidium iodide, and cell cycle analysis was performed on a Coulter Electronics Epics V flow cytometer (Coulter Electronics, Inc., Hialeah, FL.). The instrument was adjusted to achieve coefficients of variation for the nuclei of usually 3–5%. The proportion of 10,000 nuclei in G1, S, and G2-M was calculated using the Para 1 data analysis program of the flow cytometer. Figure 1 shows representative histograms obtained with the normal

**Table I** Sensitivity of normal and neoplastic human cell lines in culture to DON and acivicin

| | *Concentration (μM) of antimetabolite needed to produce 50–60% inhibition* | |
|---|---|---|
| *Cell line* | *Acivicin* | *DON* |
| IMR-90 normal fibroblast | 29.0 | 29.2 |
| A549 lung tumour | 11.6 | 9.9 |
| LX-1 lung tumour | 4.6 | 2.6 |
| SKCO-1 colon tumour | 11.6 | 5.8 |
| Redmond colon tumour | 11.6 | 4.6 |
| CX-1 colon tumour | 5.8 | 2.9 |
| CX-2 colon tumour | 17.4 | 5.8 |

The cells were preincubated for 2 h with the drug before [$^3$H]thymidine was added at 1 μ Ci $ml^{-1}$. The values listed refer to the antimetabolite concentration required to produce 50 to 60% inhibition of isotope incorporation, as outlined in the text.

Correspondence: J. Roberts.
Received 20 January 1987.

**Figure 1** DNA histograms of IMR-90 normal fibroblast and CX-1 tumour cells: effects of glutamine depletion and glutamine antimetabolites on cell cycle phase distribution. 24 h after seeding, cells were incubated either in glutamine-deficient medium, with glutaminase (0.1 IU $ml^{-1}$), acivicin or DON (6 or 30 $\mu$M). 48 h later cells were harvested and suspended in nuclei isolation medium. DNA histograms of 10,000 propidium iodide stained nuclei were obtained by flow cytometry.

fibroblast cell line (IMR-90) and one colon tumour line (CX-1). As can be seen in Figure 1 and in Table II, depletion of glutamine caused slight decreases in the S populations with concomitant increases in G1 and G2-M in all cell lines. Although treatment with 30 $\mu$M DON (or acivicin) depleted the S-phase fraction in the IMR-90 fibroblast cell line, striking S-phase blocks were observed when any of the tumour cell lines were incubated in 30 $\mu$M DON. The strikingly different responses to DON treatment by the IMR-90 fibroblasts and the tumour cell lines may be related to the slightly lower S-phase population of the IMR-90 cell line. The different responses to drug treatment could also be a reflection of the normal (but not neoplastic) cell's ability to undergo a negative pleiotypic response when the conditions in the culture medium are unfavorable to normal growth.

The results with acivicin are in agreement with earlier reports where it was shown that this analogue blocked cell cycle progression in G1 or early S-phase (Thornthwaite & Allen, 1980; Jayaram *et al.*, 1975). The effects of DON on cell cycle phase distribution have not previously been described. Both DON and acivicin have been shown to inhibit DNA synthesis by blocking *de novo* purine and pyrimidine synthesis (Weber *et al.*, 1982; Lui *et al.*, 1982; Aoki *et al.*, 1982; Levenberg *et al.*, 1957; Eidinoff *et al.*, 1958). However, our results indicating different perturbations of the cell cycle phase distribution by acivicin and DON suggest different modes of action for these glutamine antimetabolites.

For all cell lines tested the effects of acivicin and DON on cell cycle phase distribution were more pronounced if the drugs were added to glutamine-depleted medium containing dialyzed serum. Drug concentrations of 6 $\mu$M, which showed only slight perturbations in cell cycle distribution when used in media containing glutamine, exhibited much more pronounced effects in the absence of glutamine, generally showing increases in the S-phase populations (Table II, Figure 1).

Our results indicate that depletion of glutamine in the medium caused enhancement of cell cycle phase perturbations by DON and acivicin and that the normal human lung fibroblast cell line (IMR-90) was affected differently by the glutamine antimetabolites than were several human tumour cell lines. The observation that the perturbations of cell cycle phase distribution were much more pronounced when the medium lacked glutamine is therapeutically promising. These findings reinforce the therapeutic potential of administering glutamine antimetabolites in combination with a glutamine-depleting enzyme.

This investigation was supported by PHS grant CA-40446 awarded by the National Cancer Institute, NIH, USA and PCM-8212634 grant awarded by the National Science Foundation, USA.

### References

AOKI, T., SEBOLT, J. & WEBER, G. (1982). *In vivo* inactivation by acivicin of carbamoylphosphate synthetase II in rat hepatoma. *Biochem. Pharmacol.*, **31**, 927.

DUVALL, L.R. (1960). Agent data summary: 6-diazo-5-oxo-L-norleucine. *Cancer Chemother. Rep.*, **7**, 86.

EIDINOFF, M.L., KNOLL, J.E., MARANO, B. & CHEONG, L. (1958). Pyrimidine studies I. Effect of DON (6-diazo-5-oxo-L-norleucine) on incorporation of precursors into nucleic acid pyrimidines. *Cancer Res.*, **18**, 105.

HOLCENBERG, J.S. (1979). Enhanced effect of an L-glutamine antagonist, L-[αS, 5S]-α-amino-3-chloro-4,5-dihydro-5-isoxazoleacetic acid by acineto-bacter L-glutaminase-L-asparaginase. *Cancer Treat. Rep.*, **63**, 1109.

HOUCHENS, D.P., OVEJERA, A.A., SHERIDAN, M.A., JOHNSON, R.K., BOGDEN, A.E. & NEIL, G.L. (1979). Therapy of mouse tumors and human tumor xenografts with the antitumor antibiotic AT-125. *Cancer Treat. Rep.*, **63**, 473.

JAYARAM, H.N., COONEY, D.A., RYAN, J.A., NEIL, G., DION, R.L. & BONO, V.H. (1975). L-[αS, 5S]-α-amino-3-chloro-4,5-dihydro-5-isoxazoleacetic acid (NSC-163501): A new amino acid antibiotic with the properties of an antagonist of L-glutamine. *Cancer Chemother. Rep.*, **59**, 481.

LEPAGE, G.A. & LOO, T.L. (1973). Purine antagonists. In *Cancer Medicine*, Frei, E. & Holland, J.F. (eds) p. 754. Lea and Febiger: Philadelphia.

LEVENBERG, B., MELNICK, I. & BUCHANAN, J.M. (1957). Biosynthesis of the purines. XV. The effect of aza-L-serine and 6-diazo-5-oxo-L-norleucine on inosinic acid biosynthesis de novo. *J. Biol. Chem.*, **225**, 163.

LUI, M.S., KIZAKI, H. & WEBER, G. (1982). Biochemical pharmacology of acivicin in rat hepatoma cells. *Biochem. Pharmacol.*, **31**, 3469.

MITTA, S., CHOU, T.C., ROBERTS, J., STEINHERZ, P., MILLER, D. & TAN, C. (1980). Phase I trial of succinylated *Acinetobacter* glutaminase-asparaginase (SAGA) in children. *Proc. Am. Assoc. Cancer Res.*, **21**, 143.

NICHOLS, W.W., MURPHY, D.G., CRISTOFALO, V.J., TOJI, L.H., GREENE, A.E. & DWIGHT, S.A. (1977). Characterization of a new human diploid cell strain, IMR-90. *Science*, **196**, 60.

OVEJERA, A.A., HOUCHENS, D.P., CATANE, R., SHERIDAN, M.A. & MUGGIA, F.M. (1979). Efficacy of 6-diazo-5-oxi-L-norleucine and N-[N-γ-glutamyl-6-diazo-5-oxo-norleucinyl]-6-diazo-5-oxo-norleucine against experimental tumors in conventional and nude mice. *Cancer Res.*, **39**, 3220.

ROBERTS, J. (1976). Purification and properties of a highly potent anti-tumor glutaminase-asparaginase from *Pseudomonas* 7A. *J. Biol. Chem.*, **251**, 2119.

ROBERTS, J. & ROSENFELD, H. (1980). Enhancement of the antineoplastic activity of glutamine antagonists DON and AT-125 by glutaminase-asparaginase. *Proc. Am. Assoc. Cancer Res.*, **21**, 283.

ROBERTS, J., SCHMID, F.A. & ROSENFELD, H.J. (1979). Biologic and antineoplastic effects of enzyme-mediated *in vivo* depletion of L-glutamine, L-tryptophan, and L-histidine. *Cancer Treat. Rep.*, **63**, 1045.

ROSENFELD, H. & ROBERTS, J. (1981). Enhancement of antitumor activity of glutamine antagonists 6-diazo-5-oxo-L-norleucine and acivicin in cell culture by glutaminase-asparaginase. *Cancer Res.*, **41**, 1324.

SCHMID, F.A. & ROBERTS, J. (1974). Antineoplastic and toxic effects of *Acinetobacter* and *Pseudomonas* glutaminase-asparaginases. *Cancer Chemother. Rep.*, **58**, 829.

SKLAROFF, R.B., CASPER, E.S., MAGILL, G.B. & YOUNG, C.W. (1980). Phase I study of 6-diazo-5-oxo-L-norleucine (DON). *Cancer Treat. Rep.*, **64**, 1247.

THORNTHWAITE, J.T. & ALLEN, L.M. (1980). The effect of the glutamine analog, AT-125, on the cell cycle of MCF-7 and BT-20 human breast carcinoma cells using DNA flow cytometry. *Res. Com. Chem. Path. Pharmacol.*, **29**, 393.

THORNTHWAITE, J.T., SUGARBAKER, E.V. & TEMPLE, W.J. (1980). Preparation of tissues for DNA flow cytometric analysis. *Cytometry*, **1**, 229.

WEBER, G., PRAJDA, N., LUI, M.S. & 7 others (1982). Multi-enzyme targeted chemotherapy by acivicin and actinomycin. *J. Adv. Enzyme Regul.*, **20**, 75.

WEISS, G.R., McGOVERN, J.P., SCHADE, D. & KUFE, D.W. (1982). Phase I and pharmacological study of acivicin by 24-hour continuous infusion. *Cancer Res.*, **43**, 3892.